# Ring-substituted 4-Hydroxy-1*H*-quinolin-2-ones: Preparation and Biological Activity ‡

**DOI:** 10.3390/molecules14031145

**Published:** 2009-03-13

**Authors:** Josef Jampilek, Robert Musiol, Matus Pesko, Katarina Kralova, Marcela Vejsova, James Carroll, Aidan Coffey, Jacek Finster, Dominik Tabak, Halina Niedbala, Violetta Kozik, Jaroslaw Polanski, Jozef Csollei, Jiri Dohnal

**Affiliations:** 1 Zentiva k.s., U kabelovny 130, 102 37 Prague 10, Czech Republic; 2 Department of Chemical Drugs, Faculty of Pharmacy, University of Veterinary and Pharmaceutical Sciences, Palackeho 1/3, 61242 Brno, Czech Republic; 3 Institute of Chemistry, University of Silesia, Szkolna 9, 40007 Katowice, Poland; E-mail: robert.musiol@us.edu.pl (R.M.), polanski@us.edu.pl (J.P.); 4 Institute of Chemistry, Faculty of Natural Sciences, Comenius University, Mlynska dolina Ch-2, 84215 Bratislava, Slovakia; E-mail: kralova@fns.uniba.sk (M.P.); 5 Department of Biological and Medical Sciences, Faculty of Pharmacy in Hradec Kralove, Charles University in Prague, Heyrovskeho 1203, 500 05 Hradec Kralove, Czech Republic; E-mail: marcela.vejsova@faf.cuni.cz (M.V.); 6 Department of Biological Sciences, Cork Institute of Technology, Bishopstown, Cork, Ireland; E-mail: james.carroll@cit.ie (J.C.), aidan.coffey@cit.ie (A.C.)

**Keywords:** Quinolinone derivatives, Lipophilicity, OER inhibition, Spinach chloroplasts, *In vitro* antifungal activity, Structure-activity relationships.

## Abstract

In the study, a series of twelve ring-substituted 4-hydroxy-1*H*-quinolin-2-one derivatives were prepared. The procedures for synthesis of the compounds are presented. The compounds were analyzed using RP-HPLC to determine lipophilicity and tested for their photosynthesis-inhibiting activity using spinach (*Spinacia oleracea* L.) chloroplasts. All the synthesized compounds were also evaluated for antifungal activity using *in vitro* screening with eight fungal strains. For all the compounds, the relationships between the lipophilicity and the chemical structure of the studied compounds are discussed, as well as their structure-activity relationships (SAR).

## 1. Introduction

The quinoline scaffold is present in many classes of biologically-active compounds [1]. A series of compounds derived from 8-hydroxyquinoline and styrylquinoline derivatives were recently synthesized as potential HIV-1 integrase inhibitors [2,3]. These compounds show a significant similarity to some novel antifungal agents, namely homoallylamines, and therefore possess potential antifungal activity [4]. Our previous study dealing with 8-hydroxyquinoline and styrylquinoline derivatives showed that they could also possess a strong antifungal activity [5,6,7]. According to the results reported recently, some new hydroxyquinoline derivatives also possess interesting herbicidal activities [6,8,9,10,11,12,13]. Some investigated compounds also showed antineoplastic activity [14].

Photosystem II (PS II) is a multisubunit membrane protein complex, which uses light energy to oxidize water and reduce plastoquinone. Binding of herbicides to photosystem II inhibits the electron transfer from Q_A_ to Q_B_ due to competition of herbicides with plastoquinone bound at the Q_B_ site. Thus, the Q_B_ quinone-binding site of photosystem II is an important target for herbicides, including herbicides based on phenylurea moieties. It was found that a tail can be attached to the *para* position of phenylurea-type herbicides without any loss of binding, provided that the tail is hydrophobic. This indicates that the herbicides must be oriented in the Q_B_ site so that these positions point toward the natural isoprenyl tail-binding pocket that extends out of the Q_B_ site. In turn, the requirement that the tail must extend out of the Q_B_ site constrains the size of the other herbicide substituents in the pocket [15]. In addition to phenylurea-type herbicides, various other compounds possessing an amide -NHCO- moiety were also found to inhibit the photosynthetic electron transport [16,17,18,19,20,21]. Better understanding of these SAR relationships are not only important for the design of modern agricultural agents, but can also provide remarkable insights into the photosynthetic mechanisms of green cells.

Over the last three decades there has been a dramatic increase in the incidence of fungal infections, and the discovery of new drugs for the treatment of systemic mycoses is a major challenge in infectious disease research. There is an intensified need for new antifungal remedies with novel modes of action due to the rapid growth of the immunocompromised patient population, the development of resistance to the present azole therapies, and high toxicity of polyenes [22,23,24].

Compounds bearing a quinoline moiety are well known due to their broad biological activity [6]. In particular, hydroxyquinoline and its derivatives were introduced as antifungal agents in clinical practice and the novel compounds of this type are still investigated [25,26]. This paper deals with synthesis, herbicidal and antifungal activity of ring-substituted 4-hydroxy-1*H*-quinolin-2-one derivatives. All the compounds were tested for their photosynthesis-inhibiting activity (the inhibition of photosynthetic electron transport in spinach chloroplasts (*Spinacia oleracea* L.). Primary *in vitro* screening of all synthesized compounds was evaluated against eight fungal strains by means of the broth microdilution test in RPMI 1640 medium [27]. Lipophilicity (log *k*) of the compounds was determined using RP-HPLC. The procedure was performed under isocratic conditions with methanol as an organic modifier in the mobile phase using end-capped non-polar C_18_ stationary RP column. The structure-activity relationships of the compounds are also discussed.

## 2. Results and Discussion

### 2.1. Chemistry

**Scheme 1:**
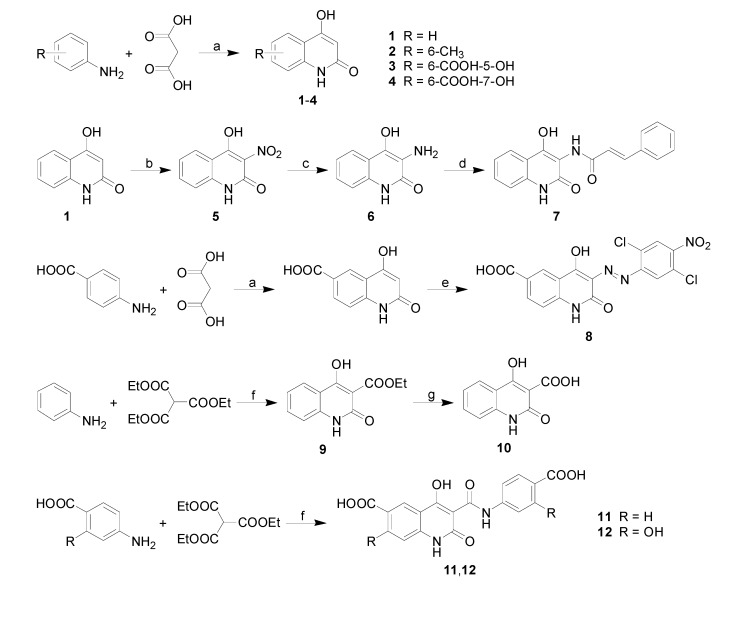
General preparation of quinoline derivatives 1-12: (a) PPA, microwave irradiation; (b) HNO_3_; (c) Sn, HCl; (d) cinnamoyl chloride; (e) (2,5-dichloro-4-nitrophenyl)diazonium chloride; (f) microwave irradiation; (g) hydrolysis.

In most of the synthesis protocols, aniline derivatives were used as the starting materials due to their convenient availability from chemical providers. Microwave assisted synthesis with malonic acid or its esters, was used to make compounds **1**-**4**. Further nitration and reduction according to established procedures were used to make compounds **5 **and **6**. Acylation of **6 **with cinnamoyl chloride provided compound **7**. Diazo derivative **8 **was made by means of a two-step synthesis from 4-aminobenzoic acid and diethyl malonate and gave 4-hydroxy-2-oxo-1,2-dihydroquinoline-6-carboxylic acid, which was coupled with the freshly prepared diazo salt derived from 4-nitro-2,5-dichloroaniline. Quinolines functionalized with carboxylic acid groups at C_(3)_ 9-12 were obtained in neat microwave assisted synthesis in moderate or good yield. Synthesis of all the above compounds is depicted in Scheme 1.

### 2.2. Lipophilicity

Hydrophobicities (log *P*/Clog *P* values) of the compounds **1**-**12** were calculated using two commercially available programs and also measured by means of the reversed phase high performance liquid chromatography (RP-HPLC) method for lipophilicity measurement. The procedure was performed under isocratic conditions with methanol as an organic modifier in the mobile phase using an end-capped non-polar C_18_ stationary RP column. The capacity factors *k* were determined and subsequent log *k* values were calculated. 

**Figure 1:**
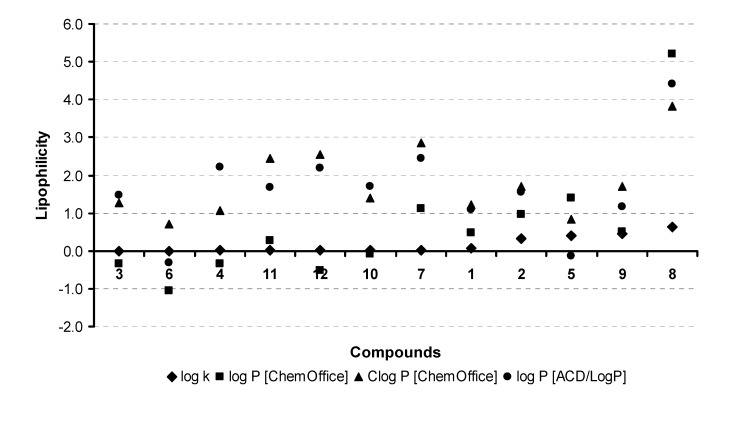
Comparison of the computed log *P*/Clog *P* values using the two programs with the calculated log *k* values. The discussed compounds 1-12 are ordered according to the log *k* values increase.

The results are summarized in Table 1 and illustrated in Figure 1. The results obtained with all the compounds show that the experimentally-determined lipophilicities (log *k* values) are lower than those indicated by the calculated log *P*/Clog *P*, as shown in Figure 1, indicating relatively poor correlation between the experimentally-determined log *k* values and the calculated values. As expected, compound **8** showed the highest lipophilicity, while compound **3 **possessed the lowest hydrophobicity, which was unexpected. Compound **7** showed less hydrophobicity contrary to all the results of the lipophilicity calculated by software. Comparing the lipophilicity data log *k* of both position analogues **3** and **4**, it can be stated that the 7-hydroxy derivative **4** possessed higher hydrophobicity than 5-hydroxy analogue **3**. The salicylic acid derivative **12** showed higher lipophilicity than benzoic derivative **11**. These facts are caused by intramolecular interactions [28].

**Table 1:** Comparison of the calculated lipophilicities (log *P*/Clog *P*) with the determined log *k* values.

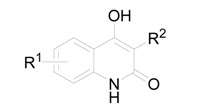					
Comp.	R^1^	R^2^	log *k*	log *P*/Clog *P* ChemOffice	log *P* ACD/LogP
**1**	H	H	0.0664	0.49 / 1.216	1.10 ± 0.75
**2**	6-CH_3_	H	0.3307	0.97 / 1.715	1.56 ± 0.75
**3**	6-COOH-5-OH	H	0.0002	-0.34 / 1.261	1.47 ± 0.75
**4**	6-COOH-7-OH	H	0.0080	-0.34 / 1.070	2.22 ± 0.75
**5**	H	-NO_2_	0.4052	1.39 / 0.836	-0.14 ± 1.00
**6**	H	-NH_2_	0.0004	-1.06 / 0.719	-0.32 ± 1.00
**7**	H	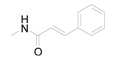	0.0128	1.11 / 2.848	2.45 ± 1.00
**8**	6-COOH	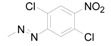	0.6394	5.22 / 3.840	4.41 ± 1.00
**9**	H	-COOC_2_H_5_	0.4595	0.51 / 1.694	1.17 ± 0.75
**10**	H	-COOH	0.0118	-0.09 / 1.409	1.71 ± 0.35
**11**	6-COOH	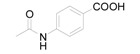	0.0081	0.27 / 2.445	1.67 ± 1.00
**12**	6-COOH-5-OH	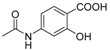	0.0093	-0.51 / 2.543	2.20 ± 1.00

### 2.3. Oxygen evolution rate inhibition in spinach chloroplasts

All compounds were evaluated for their *in vitro* herbicidal efficiency. The results are listed in Table 2. Quinoline derivatives **1**-**12** showed a wide range of activity related to inhibition of oxygen evolution rate (OER) in spinach chloroplasts. Two compounds showed interesting IC_50_ (half maximal inhibitory concentration) values: 126 µmol/L (**8**) and 157 µmol/L (**2**); nevertheless the studied activity of all the other compounds was very low.

Due to the moderate and/or low activity of compounds **1**-**12**, it is difficult to determine simple structure-activity relationships. However some interesting observations can be made: in the case of compound **1**, an unsubstituted structure did not have any effect on OER in chloroplasts. The remaining compounds could be divided into two groups according to their chemical structure. Group 1 includes compounds **2**-**4**, **8**, **11** and **12**, and Group 2 compounds **5-7**, **9** and **10**.

**Figure 2:**
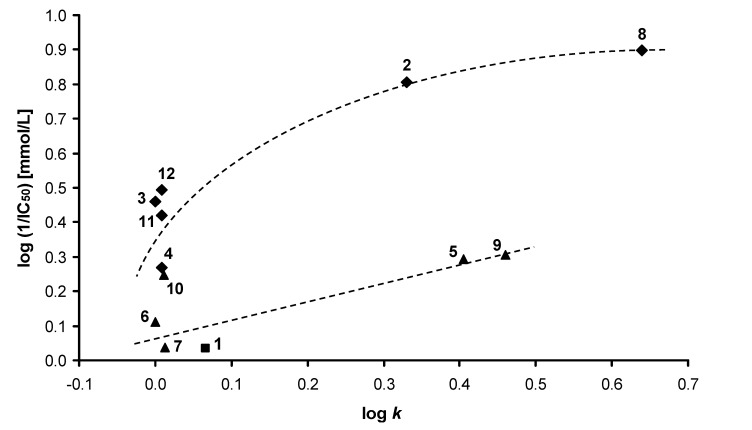
Relationships between the OER inhibition log (1/IC_50_) [mmol/L] in spinach chloroplasts and lipophilicity (log *k*) of the studied compounds **1**-**12**.

Group 1 showed higher biological activity than Group 2. The activity related to OER inhibition seems to be positively influenced by substitution of ring B: especially the C_(6)_ position (see compounds **2-4**, **11**, **12**). Comparison of the OER-inhibiting activities of compounds **2-4**, **8**, **11** and **12** also indicated that the lipophilicity increase is connected with the quasi-parabolic increase of biological activity (Figure 2).

**Table 2:** IC_50_ values related to OER inhibition in spinach chloroplasts in comparison with 3-(3,4-dichlorophenyl)-1,1-dimethylurea (DCMU) standard and *in vitro* antifungal activity (IC_80_) of compounds **1-12** compared with fluconazole (FLU) standard.

Comp.	OER inhibition	MIC/IC_80_ [µmol/L]							
		CA	CT	CK	CG	TB	AF	AC	TM
	IC_50_ [μmol/L]	*24h*	*24h*	*24h*	*24h*	*24h*	*24h*	*24h*	*72h*
		*48h*	*48h*	*48h*	*48h*	*48h*	*48h*	*48h*	*120h*
**1**	925	>500	>500	>500	>500	>500	>500	>500	>500
		>500	>500	>500	>500	>500	>500	>500	>500
**2**	157	>500	>500	>500	>500	>500	>500	>500	>500
		>500	>500	>500	>500	>500	>500	>500	>500
**3**	346	125	500	>500	250	250	>500	>500	>500
		125	>500	>500	250	>500	>500	>500	>500
**4**	538	15.62	500	>500	62.50	62.50	500	>500	>500
		62.50	>500	>500	250	>500	>500	>500	>500
**5**	510	>500	>500	>500	>500	>500	>500	>500	>500
		>500	>500	>500	>500	>500	>500	>500	>500
**6**	775	>500	>500	>500	>500	>500	>500	>500	>500
		>500	>500	>500	>500	>500	>500	>500	>500
**7**	916	>125	>125	>125	>125	>125	>125	>125	>125
		>125	>125	>125	>125	>125	>125	>125	>125
**8**	126	31.25	250	250	250	250	125	62.50	62.50
		125	>250	250	>250	>250	250	250	125
**9**	494	>500	>500	>500	>500	>500	>500	>500	>500
		>500	>500	>500	>500	>500	>500	>500	>500
**10**	567	>500	>500	>500	>500	>500	>500	>500	>500
		>500	>500	>500	>500	>500	>500	>500	>500
**11**	380	>500	>500	>500	>500	>500	>500	>500	>500
		>500	>500	>500	>500	>500	>500	>500	>500
**12**	321	62.50	500	>500	125	125	500	500	500
		125	>500	>500	250	>500	>500	>500	>500
**DCMU**	1.9	-	-	-	-	-	-	-	-
**FLU**	-	0.06	0.12	3.91	0.98	0.24	>125	>125	1.95
		0.12	>125	15.62	3.91	0.48	>125	>125	3.91

It is noteworthy that there are great differences in OER inhibition levels caused by positional isomers **3** (6-COOH-5-OH) and **4** (6-COOH-7-OH). Introducing a further phenolic moiety in compound **12** (salicylic derivative) positively influenced OER inhibition. The higher inhibitory effect of compound 8 compared with compound **2** may have resulted from higher lipophilicity (easier penetration of the compound through cell walls) and/or redox properties of the nitro moiety of the 2,5-dichloro-4-nitrophenyldiazenyl substituent.

Generally, Group 2 compounds only caused slight inhibition of OER; nevertheless compounds **5** and **9** were approximately twice as effective as compound **1**. All these compounds possess the substituted position C_(3)_ of ring A, which caused the decrease in OER inhibition compared with Group 1 compounds. The most active compound from Group 2 was the ester **9**.

### 2.4. In vitro antifungal susceptibility testing

All the compounds were tested for their *in vitro* antifungal activity. Compounds **1-3, 5-7, 9-11** did not show any activity and compounds **4**, **8** and **12** showed only a moderate activity, especially against *Candida albicans* ATCC 44859. Compound **4** showed medium activity against *Candida glabrata* 20/I, and compound **8** against *Trichophyton mentagrophytes* 445. The activities of the compounds are shown in Table 2.

Generally, it can be stated that *in vitro* antifungal activity depends on heteroaromatic ring A. Hydrogenation of ring A and introduction of keto group resulted in the loss of the antifungal effect compared with hydroxyquinoline derivatives [6,7]. Substitution of the C_(3)_ position by various moieties did not have a significant influence on the activity. Nevertheless salicylic acid derivative **12** showed a higher activity compared with benzoic derivative **11**, probably due to the substitution of the C_(3)_´ position by phenolic moiety.

Unsubstituted ring B or C_(6)_ substitution by a methyl moiety did not results in any activity. Substitution of phenyl ring B by 6-COOH (compounds **3**, **4**, **8** and **11, 12**) caused the activity to increase. Position of the phenolic moiety seems to be a very important parameter for antifungal activity. While a 6-COOH-5-OH substitution pattern (compound **3**) did not show any activity increase, introduction of 6-COOH along with a 7-OH moiety (compound **4**) increased the activity significantly. The antifungal activity of compounds **8** and **12** was connected with 2,4-dichloro-4-nitrophenyldiazenyl and 3-(4-carboxy-3-hydroxyphenylcarbamoyl) substituents, respectively. According to the results, it can be assumed that lipophilicity is only of secondary importance for antifungal activity.

## 3. Conclusions

A series of twelve ring-substituted 4-hydroxy-1*H*-quinolin-2-one derivatives were prepared and characterized. All the prepared quinoline derivatives were analyzed using a RP-HPLC method for the lipophilicity measurement and their lipophilicity was determined. The prepared compounds were tested for their photosynthesis-inhibiting activity (the inhibition of photosynthetic electron transport in spinach chloroplasts (*Spinacia oleracea* L.) and for their antifungal activity. (*E*)-3-[(2,5-Dichloro-4-nitrophenyl)diazenyl]-4-hydroxy-2-oxo-1,2-dihydroquinoline-6-carboxylic acid (**8**) showed the highest OER inhibition activity and 4,7-dihydroxy-2-oxo-1,2-dihydroquinoline-6-carboxylic acid (**4**) and compound **8** possessed the highest *in vitro* antifungal activity within the series.

## 4. Experimental

### 4.1. General

All reagents were purchased from Aldrich. Kieselgel 60, 0.040-0.063 mm (Merck, Darmstadt, Germany) was used for column chromatography. TLC experiments were performed on alumina-backed silica gel 40 F254 plates (Merck, Darmstadt, Germany). The plates were illuminated under UV (254 nm) and evaluated in iodine vapour. The melting points were determined on Boetius PHMK 05 (VEB Kombinat Nagema, Radebeul, Germany) and are uncorrected. Elemental analyses were carried out on an automatic Perkin-Elmer 240 microanalyser (Boston, USA). The purity of the final compounds was checked by the HPLC separation module Waters Alliance 2695 XE (Waters Corp., Milford, MA, U.S.A.). The detection wavelength 210 nm was chosen. The peaks in the chromatogram of the solvent (blank) were deducted from the peaks in the chromatogram of the sample solution. The purity of individual compounds was determined from the area peaks in the chromatogram of the sample solution. UV spectra (λ, nm) were determined on a Waters Photodiode Array Detector 2996 (Waters Corp., Milford, MA, U.S.A.) in ca 6×10^-4^ mol methanolic solution and log ε (the logarithm of molar absorption coefficient ε) was calculated for the absolute maximum λ_max_ of individual target compounds. Infrared spectra were recorded in a Smart MIRacle™ ATR ZnSe for Nicolet™ 6700 FT-IR Spectrometer (Thermo Scientific, U.S.A.). Spectra are corrigated. All ^1^H NMR spectra were recorded on a Bruker AM-500 (499.95 MHz for ^1^H), Bruker BioSpin Corp., Germany. Chemicals shifts are reported in ppm (δ) to internal Si(CH_3_)_4_, when diffused easily exchangeable signals are omitted.

*4-Hydroxyquinolin-2(1H)-one* (**1**). Preparation of PPA: P_2_O_5_ (287.9 g) was added to 85% phosphoric acid (200 g, 118.4 mL) under stirring and microwave heating. The mixture was then heated for 15 min. Aniline (7 mL, 5 mmol) and malonic acid (5.2 g, 5 mmol) were thoroughly mixed with 20 g PPA and heated under stirring in microwave reactor at 400 W during 2×20 min with 5 min interval. The temperature reached 210 °C. Then the mixture was poured into crushed ice and the beige solid was filtered and purified by extraction with EtOH and a white crystalline compound was obtained [29]. Yield 35%; Mp 340 °C; HPLC purity 97.12%; UV (nm), λ_max_/log ε: 231.3/3.51; IR (cm^-1^): 3618, 1180 (OH), 3043 (=CH-), 1670 (lactam), 1650 (C=O), 1593 (Ph), 1522 (NH).

*4-Hydroxy-6-methylquinolin-2(1H)-one* (**2**). The product was obtained according to the previously described procedure [30,31] as a light brown crystalline compound. Yield 35%; Mp 320 °C; HPLC purity 97.72%; UV (nm), λ_max_/log ε: 232.4/3.55; IR (cm^-1^): 3618, 1180 (OH), 3044 (=CH-), 2965, 1379 (CH_3_), 1668 (lactam), 1652 (C=O), 1592 (Ph), 1522 (NH).

*4,5-Dihydroxy-2-oxo-1,2-dihydroquinoline-6-carboxylic acid* (**3**). Naphthalene (15.4 g, 0.12 mol) and malonic acid (18.7 g, 0.18 mol) were melted with stirring under temperature control (<150 °C) to avoid decarboxylation of the acid. POCl_3_ (32.9 g, 0.36 mol) was then added dropwise over 30 min and *p*-aminosalicylic acid (15.3 g, 0.1 mol) was then added. The resulting mixture was heated for 30 min and allowed to cool. Water (100 mL) was added to the warm mixture and the solution was made alkaline to pH 9 with 20% NaOH. After cooling on ice precipitated naphthalene, it was filtered and the filtrate was acidified to pH 2. The product was filtered again and crystallized from acetic acid as a bright yellow crystalline compound. Yield 36%; Mp 250 °C; Anal. calc. for C_10_H_7_NO_5_ (221.16): C 54.31%, H 3.19%; found: C 54.51%, H 4.11%; HPLC purity 98.74%; UV (nm), λ_max_/log ε: 244.2/3.54; IR (cm^-1^): 3620, 1180 (OH), 3045 (=CH-), 2950, 1690 (acid), 1672 (lactam), 1650 (C=O), 1598 (Ph), 1523 (NH), 1329, 1198 (OH_phenol_); ^1^H-NMR (DMSO-*d_6_*) δ: 5.65 (s, 1H), 6.60 (d, *J*=8.3 Hz, 1H), 7.80 (d, *J*=8.3 Hz, 1H), 11.3 (bs, 1H), 12.20 (bs, 1H).

*4,7-Dihydroxy-2-oxo-1,2-dihydroquinoline-6-carboxylic acid* (**4**). The product was obtained as an isomer of **3** during its synthesis. Isolated by fractional crystalization as a white crystalline compound. Yield 36%; Mp 250 °C; Anal. calc. for C_10_H_7_NO_5_ (221.16): C 54.31%, H 3.19%; found: C 54.09%, H 3.52%; HPLC purity 98.51%; UV (nm), λ_max_/log ε: 243.0/3.54; IR (cm^-1^): 3618, 1181 (OH), 3043 (=CH-), 2948, 1693 (acid), 1670 (lactam), 1651 (C=O), 1599 (Ph), 1521 (NH), 1328, 1200 (OH_phenol_); ^1^H-NMR (DMSO-*d_6_*) δ: 5.60 (s, 1H), 6.67 (s, 1H), 8.25 (s, 1H).

*4-Hydroxy-3-nitroquinolin-2(1H)-one* (**5**). The product was obtained according to the described nitration procedure [32] as a yellow crystalline compound. Yield 71%; Mp 252-255 °C; HPLC purity 99.72%; UV (nm), λ_max_/log ε: 336.8/3.57; IR (cm^-1^): 3620, 1181 (OH), 1712 (C=O), 1682 (lactam), 1622 (C=C_cycle_), 1595 (Ph), 1547 (NO_2_), 1525 (NH).

*3-Amino-4-hydroxyquinolin-2(1H)-one* (**6**). Compound **6** (2.0 g, 0.0097 mol) and tin powder (3.8 g, 0.032 mol) were stirred with 36% HCl (8.1 mL). The mixture was warmed at 80-90 °C for 30 min. The brown solution was cooled to room temperature and filtered. The filtrate was alkalized with NH_3(aq)_ and warmed for 20 min. Then Celite (1.3 g) was added and filtered. The solid was washed thoroughly with hot water (80 °C). The combined filtrates were concentrated and acidified. After cooling a white crystalline compound was obtained. Yield 85%; Mp 300 °C [33]; HPLC purity 91.99%; UV (nm), λ_max_/log ε: 232.8/3.53; IR (cm^-1^): 3620, 1181 (OH), 3312, 1618 (NH_2_), 1670 (lactam), 1650 (C=O), 1625 (C=C_cycle_), 1598 (Ph), 1523 (NH).

*(2E)-N-(4-Hydroxy-2-oxo-1,2-dihydroquinolin-3-yl)-3-phenylprop-2-enamide* (**7**). Compound **7** (0.018 g, 0.001 mol) was mixed with water (5 mL), Et_2_O (5 mL) and sodium bicarbonate (0.3 g). The resulted mixture was stirred in an ice bath (-3 °C) and 10 mL of Et_2_O solution of cinamoyl chloride (0.017 g, 0.001 mol) was added slowly. The resulting mixture was stirred at room temperature for 2 days, cooled in fridge and filtered. Et_2_O was added to the solid and dried. A white crystalline compound was obtained. Yield 50%; Mp 145 °C; Anal. calc. for C_18_H_14_N_2_O_3_+H_2_O (324.33): C 66.66%, H 4.97%; found: C 66.54%, H 5.27%; HPLC purity 99.79%; UV (nm), λ_max_/log ε: 263.1/3.51; IR (cm^-1^): 3620, 1180 (OH), 3035 (CH_arom_), 1670 (lactam), 1650 (C=O), 1648 (amide), 1628 (C=C_cycle_), 1618, 974 (C=C), 1599 (Ph), 1525 (NH); ^1^H-NMR (DMSO-*d_6_*) δ: 3.30 (s, 1H), 6.50 (d, *J*=16.2 Hz, 2H), 7.10 (s, 1H), 7.38 (m, 9H), 7.5 (s, 1H).

*(E)-3-[(2,5-Dichloro-4-nitrophenyl)diazenyl]-4-hydroxy-2-oxo-1,2-dihydroquinoline-6-carboxylic acid* (**8**). 4-Hydroxy-2-oxo-1,2-dihydroquinoline-6-carboxylic acid was obtained as compound **3** from 4-aminobenzoic acid and used without thorough purification in further synthesis as follows. IR (cm^-1^): 3618, 1179 (OH), 3043 (=CH-), 2948, 1686 (acid), 1677 (lactam), 1650 (C=O), 1599 (Ph), 1523 (NH); ^1^H-NMR (DMSO-*d_6_*) δ: 7.7 (s, 1H), 7.9 (m, 3H), 10.43 (s, 1H), 10.47 (s, 1H), 12.7 (s, 1H). 2,5-Dichloro-4-nitroaniline (0.92 g) was dissolved in 2:1 Et_2_O/EtOH , then 15% HCl (0.91 mL) was added to this solution and the mixture was cooled to 5 °C. NaNO_2_ (0.4 g, 5.7 mmol) and 4-hydroxy-2-oxo-1,2-dihydroquinoline-6-carboxylic acid (1.0 g, 5.7 mmol) was added slowly under the temperature of 5 °C and pH<7 (15% HCl). The resulting mixture was left in ice overnight. The precipitated solid was then filtered and crystallized from Et_2_O/EtOH. A reddish crystalline compound was obtained. Yield 64%; Mp 340 °C; Anal. calc. for C_16_H_8_Cl_2_N_4_O_6_ (423.16): C 45.41%, H 1.91%; found: C 45.26%, H 2.24%; HPLC purity 96.39%; UV (nm), λ_max_/log ε: 271.4/3.61; IR (cm^-1^): 3616, 1180 (OH), 3030 (CH_arom_), 2950, 1680 (acid), 1670 (lactam), 1655 (C=O), 1630 (C=C_cycle_), 1614 (N=N), 1598 (Ph), 1543 (NO_2_), 1520 (NH); ^1^H-NMR (DMSO-*d_6_*) δ: 5.70 (s, 1H), 7.10-7.60 (m, 3H), 11.10 (s, 1H), 11.30 (s, 1H).

*Ethyl 4-hydroxy-2-oxo-1,2-dihydroquinoline-3-carboxylate* (**9**). Aniline (0.46 mL, 0.005 mol) and triethyl methanetricarboxylate (2.12 mL, 0.01 mol) were heated in microwave reactor for 8 min at 60% power level. The mixture was then cooled to room temperature and 7 mL of Et_2_O was added. The crude product was crystallized from MeOH. A white crystalline compound was obtained. Yield 50%; Mp 116-120 °C; Anal. calc. for C_12_H_11_NO_4_ (233.22): C 61.80%, H 4.75%; found: C 61.65%, H 4.39%; HPLC purity 95.01%; UV (nm), λ_max_/log ε: 244.2/3.59; IR (cm^-1^): 3620, 1180 (OH), 2958 (CH_3_), 2925 (CH_2_), 1680 (lactam), 1638 (C=O), 1630 (C=C_cycle_), 1598 (Ph), 1520 (NH), 1191 (C=O_ester_); ^1^H-NMR (DMSO-*d_6_*) δ: 1.19 (t, 3H), 4.17 (q, 2H), 4.70 (s, 1H), 7.09 (t, 2H), 7.32 (t, 1H), 7.52 (d, *J*=8.5 Hz, 1H), 10.30 (t, 1H).

*4-Hydroxy-2-oxo-1,2-dihydroquinoline-3-carboxylic acid* (**10**). The product was obtained according to the described procedure [34,35] as a white crystalline compound. Yield 99%; Mp 225 °C; HPLC purity 99.51%; UV (nm), λ_max_/log ε: 250.1/3.52; IR (cm^-1^): 3621, 1182 (OH), 2965, 1670 (acid), 1679 (lactam), 1646 (C=O), 1629 (C=C_cycle_), 1599 (Ph), 1525 (NH).

*3-(4-Carboxyphenylcarbamoyl)-4-hydroxy-2-oxo-1,2-dihydroquinoline-6-carboxylic acid* (**11**). 4-Aminobenzoic acid (0.7 g, 0.005 mol) was mixed with triethyl methanetricarboxylate (2.12 mL, 0.01 mol) and heated in microwave reactor at 50% of power during 15 min and 3 min at 90%. The temperature reached 231 °C during heating. Et_2_O was added to the cooled mixture and the precipitate was washed with hot (55 °C) MeOH to obtain the pure product as a yellow crystalline compound. Yield 62%; Mp 340-350 °C; Anal. calc. for C_18_H_12_N_2_O_7_ (368.29): C 58.70%, H 3.28%; found: C 58.09%, H 3.54%; HPLC purity 97.52%; UV (nm), λ_max_/log ε: 251.3/3.53; IR (cm^-1^): 3621, 1180 (OH), 3034 (CH_arom_), 2970, 1689 (acid), 1680 (lactam), 1642 (C=O), 1635 (C=C_cycle_), 1630 (amide), 1599 (Ph), 1520 (NH); ^1^H-NMR (DMSO-*d_6_*) δ: 7.41 (d, *J*=8.5 Hz, 1H), 7.70 (d, *J*=9.1 Hz, 2H), 7.90 (d, *J*=9.1 Hz, 2H), 8.15 (d, *J*=8.5 Hz, 1H), 8.50 (s, 1H), 12.40 (s, 1H), 12.95 (s, 1H), 16 (s, 1H).

*3-(4-Carboxy-3-hydroxyphenylcarbamoyl)-4-hydroxy-2-oxo-1,2-dihydroquinoline-6-carboxylic acid* (**12**). 4-Aminosalicylic acid (0.7 g, 0.005 mol) was mixed with triethyl methanetricarboxylate (2.12 mL, 0.01 mol) and heated in microwave reactor at 50% of power for 15 min and 3 min at 90%. The temperature reached 230 °C during heating. Et_2_O was added to the cooled mixture and the precipitate was washed with hot (55 °C) MeOH to obtain the pure product as a yellow crystalline compound. Yield 20%; Mp 350 °C; Anal. calc. for C_18_H_12_N_2_O_9_ (400.29): C 54.01%, H 3.02%; found: C 54.05%, H 9.94%; HPLC purity 96.42%; UV (nm), λ_max_/log ε: 256.0/3.53; IR (cm^-1^): 3620, 1179 (OH), 3035 (CH_arom_), 2972, 1688 (acid), 1680 (lactam), 1640 (C=O), 1633 (C=C_cycle_), 1632 (amide), 1600 (Ph), 1521 (NH), 1329, 1199 (OH_phenol_); ^1^H-NMR (DMSO-*d_6_*) δ: 7.43 (d, *J*=8.5 Hz, 2H), 7.7 (s, 1H), 7.9 (m, 3H), 10.43 (s, 1H), 10.47 (s, 1H), 12.7 (s, 1H) 16.0 (s, 1H).

### 4.2. Lipophilicity HPLC determination (capacity factor k / calculated log k)

The HPLC separation module Waters Alliance 2695 XE and Waters Photodiode Array Detector 2996 (Waters Corp., Milford, MA, U.S.A.) were used. A Symmetry^®^ C_18_ 5 μm, 4.6 × 250 mm, Part No. WAT054275, (Waters Corp., Milford, MA, U.S.A.) chromatographic column was used. The HPLC separation process was monitored by Millennium32^®^ Chromatography Manager Software, Waters 2004 (Waters Corp., Milford, MA, U.S.A.). The mixture of MeOH p.a. (55.0%) and H_2_O-HPLC – Mili-Q Grade (45.0%) was used as a mobile phase. The total flow of the column was 0.9 mL/min, injection 30 μL, column temperature 30 °C and sample temperature 10 °C. The detection wavelength 210 nm was chosen. The KI methanolic solution was used for the dead time (T_D_) determination. Retention times (t_R_) were measured in minutes. The capacity factors *k* were calculated using the Millennium32^®^ Chromatography Manager Software according to formula *k* = (t_R_ - t_D_) / t_D_, where t_R_ is the retention time of the solute, whereas t_D_ denotes the dead time obtained via an unretained analyte. Log *k*, calculated from the capacity factor *k*, is used as the lipophilicity index converted to log *P* scale. The log *k* values of the individual compounds are shown in Table 1.

### 4.3. Lipophilicity calculations

Log *P*, *i.e.* the logarithm of the partition coefficient for *n-*octanol/water, was calculated using the programs CS ChemOffice Ultra ver. 9.0 (CambridgeSoft, Cambridge, MA, U.S.A.) and ACD/LogP ver. 1.0 (Advanced Chemistry Development Inc., Toronto, Canada). Clog *P* values (the logarithm of *n*-octanol/water partition coefficient based on established chemical interactions) were generated by means of CS ChemOffice Ultra ver. 9.0 (CambridgeSoft, Cambridge, MA, U.S.A.) software. The results are shown in Table 1.

### 4.4. Study of inhibition of oxygen evolution rate (OER) in spinach chloroplasts

Chloroplasts were prepared from spinach (*Spinacia oleracea* L.) according to Masarovicova and Kralova [36]. The inhibition of photosynthetic electron transport (PET) in spinach chloroplasts was determined spectrophotometrically (Genesys 6, Thermo Scientific, U.S.A.) using an artificial electron acceptor 2,6-dichlorophenol-indophenol (DCPIP) according to Kralova *et al*. [37] and the rate of photosynthetic electron transport was monitored as a photoreduction of DCPIP. The measurements were carried out in phosphate buffer (0.02 mol/L, pH 7.2) containing sucrose (0.4 mol/L), MgCl_2_ (0.005 mol/L) and NaCl (0.015 mol/L). The chlorophyll content was 30 mg/L in these experiments and the samples were irradiated (~100 W/m^2^) from 10 cm distance with a halogen lamp (250 W) using a 4 cm water filter to prevent warming of the samples (suspension temperature 22 °C). The studied compounds were dissolved in DMSO due to their limited water solubility. The applied DMSO concentration (up to 4%) did not affect the photochemical activity in spinach chloroplasts. The inhibitory efficiency of the studied compounds was expressed by IC_50_ values, *i.e.* by molar concentration of the compounds causing 50% decrease in the oxygen evolution rate relative to the untreated control. The comparable IC_50_ value for a selective herbicide 3-(3,4-dichlorophenyl)-1,1-dimethylurea, DCMU (Diurone^®^) was about 1.9 μmol/L [38]. The results are summarized in Table 2.

### 4.4. In vitro antifungal susceptibility testing

The broth microdilution test [27,39] was used for the assessment of *in vitro* antifungal activity of the synthesized compounds against *Candida albicans* ATCC 44859 (CA), *Candida tropicalis* 156 (CT), *Candida krusei* ATCC 6258 (CK), *Candida glabrata* 20/I (CG), *Trichosporon beigelii* 1188 (TB), *Aspergillus fumigatus* 231 (AF), *Absidia corymbifera* 272 (AC), and *Trichophyton mentagrophytes* 445 (TM). Fluconazole (FLU) was used as the standard of a clinically used antimycotic drug. The procedure was performed with twofold dilution of the compounds in RPMI 1640 (Sevapharma a.s., Prague, Czech Republic) buffered to pH 7.0 with 0.165 mol of 3-morpholino-propane-1-sulfonic acid (MOPS, Sigma, Germany). The final concentrations of the compounds ranged from 500 to 0.975 μmol/l. Drug–free controls were included. The MIC was defined as an 80% or greater (IC_80_) reduction of growth in comparison with the control. The values of MICs were determined after 24 and 48 h of static incubation at 35 °C. For *T. mentagrophytes*, the final MICs were determined after 72 and 120 h of incubation. The results are summarized in Table 2.
