# *Publisher's Note* - Pagination Error, *Molecules* 2009, *14*(3)

**DOI:** 10.3390/molecules14031134

**Published:** 2009-09-10

**Authors:** Shu-Kun Lin

**Affiliations:** Molecular Diversity Preservation International (MDPI), Kandererstrasse 25, CH-4057 Basel, Switzerland; E-mail: lin@mdpi.org

*Publisher's Note* added on 10 September 2009: There is a pagination error in *Molecules* **2009**, *14*(3) with pages 1134-1144 missing. Thus, pages 1134-1144 are taken as a blank pages.

